# Comparative infection of autologous primary T cells and monocytes derived DCs using cell-free virus preparation, viral biofilm, pseudotyped-virus or viral synapse

**DOI:** 10.1186/1742-4690-11-S1-P99

**Published:** 2014-01-07

**Authors:** Sandrine Alais, Hélène Dutartre, Renaud Mahieux

**Affiliations:** 1 Oncogenèse Rétrovirale, label « Ligue Nationale Contre le Cancer », CIRI, Labex Ecofect, INSERM U1111-CNRS UMR5308, Université Lyon-1, Ecole Normale Supérieure, Lyon, Cedex 07, France

## 

HTLV-1 infected T cells and blood dentritic cells (DCs) can be found in HTLV-1 carriers. It is currently believed that T cell infection requires cell-cell contact, while DCs could also be infected with cell-free virus. However, a comparative study using different modes of viral preparation for infecting human primary autologous T and DC cells has not been performed. Supernatant from HTLV-1 chronically infected T cells, cell conjugates between HTLV-1 infected cells and target cells, or purified viral biofilm were used side-by-side to infect autologous human primary lymphocytes and monocytes-derived dendritic cells. To monitor the early steps of viral infection, we also used pseudotyped viruses carrying either the HTLV-1 or the VSV envelopes. Infection was then followed by flow cytometry, ELISA, immunofluorescence, real-time PCR and alu-PCR. Our first data show that, depending on the source of viruses, lymphocytes have distinct susceptibility to HTLV-1 infection. The impact of the viral source in the spread of HTLV-1 infection will be discussed.

